# Current practices of plastic waste management, environmental impacts, and potential alternatives for reducing pollution and improving management

**DOI:** 10.1016/j.heliyon.2024.e40838

**Published:** 2024-11-29

**Authors:** Md Atik Fayshal

**Affiliations:** Department of Civil, Construction, and Environmental Engineering, North Dakota State University (NDSU), Fargo, ND, 58108, USA

**Keywords:** Global plastic production, Environmental impacts, Current management practices, Challenges and future prospects

## Abstract

Plastic products are indispensable across various applications, yet their disposal poses significant environmental hazards, such as groundwater contamination, soil degradation, and marine ecosystem threats, impacting both human health and ecological balance. Key issues include rapid development and population growth, inadequate technical skills for hazardous waste management, insufficient infrastructure for recycling, and a general lack of awareness regarding regulations. This study offers a comprehensive analysis of plastic waste generation, its sources, current management practices, and its environmental impacts, along with mitigation measures. Globally, predictions indicate that plastic production could exceed 650 million tons by 2050, representing a staggering more than 22000 % increase compared to 1950, highlighting the pressing need for action. Moreover, due to their chemical structures and prolonged degradation periods, plastic waste (PW) can lead to cancer, nervous system damage, rapid genetic changes, and metabolic disorders in humans. In that scenario, waste-to-energy and product conversion strategies through thermochemical conversion techniques can be a double-edged solution by minimizing waste along with providing value-added products. Also, using alternatives to plastic, including bio-plastics, stainless steel, glass, platinum silicon, wood, bamboo, cardboard, paper, cotton, pottery, ceramics, and more, can be a viable pathway for proper PW management. The successful incorporation of the proposed alternative products holds promise for improving the overall management of plastic waste. Additionally, this study highlights sustainable waste management practices and outlines the primary challenges in implementing effective strategies to reduce the negative impact of plastic waste.

## 1 Introduction

Plastics have replaced many traditional materials in consumer goods due to their lightweight nature, durability, resistance to corrosion by most chemicals, versatility in applications, ease of processing, and cost-effectiveness. However, these advantages come with significant environmental drawbacks. Plastic pollution has gathered significant international attention, ranking as the world's third major contributor to waste. Additionally, recent studies have shown that oil-based plastics are highly resistant to biodegradation, which leads to their accumulation in the environment [1]. This challenge is compounded by rapid industrialization and population growth, resulting in an inevitable surge in both organic and inorganic waste in cities. The global increase in plastic waste is driven by population growth and rising per capita consumption [2]. Moreover, Inadequate disposal practices and a disposable mentality exacerbate the buildup of plastic waste, leading to the depletion of valuable resources and contamination of both terrestrial and aquatic ecosystems [1,3].

Research indicates that the current global waste generation is around 1.7–1.9 billion metric tons per year, the figure expected to climb to 27 billion metric tons per year by 2050. Of this waste, almost one-third is projected to originate from Asia alone [4]. Approximately 50–70 % of this extensive waste output is collected for disposal, with unmanaged landfilling making up 15 % of the gathered waste [5,6]. The environmental impact of this waste is exacerbated by the presence of plastic litter, which accounts for nearly 5 % of municipal solid waste [7]. This situation is further complicated by the high per capita consumption of plastic products, particularly in North America and Asia, where people consume around 120 kg of plastic-driven products per capita, respectively. The non-biodegradability of plastics complicates their disposal, leading to significant environmental challenges [8].

For several years, Asia has been the largest consumer of polymers, producing 30 % of all plastic waste [9]. From 1992 to 2016, China served as the destination for 45 % of the globe's plastic waste, leading to the widespread dispersal of immense quantities of plastic waste following China's import ban. This dispersion is anticipated to accumulate to 111 million metric tons by 2030 [10]. The ban has caused a significant shift in the global plastic recycling system, causing a state of panic and requiring rapid adaptation [11]. Even though numerous countries acknowledge the significance of recycling and tapping into domestic plastic waste streams, they face a shortage of the required industrial infrastructure and capacity [12]. As a result, a large amount of plastic waste was exported to other Asian countries such as Indonesia, Vietnam, Malaysia, and the Philippines, and Turkey has also emerged as a new plastic waste recycling market for some European countries [13,14]. According to recent projections, global municipal solid waste generation is expected to increase from approximately 2.3 billion metric tons in 2023 to 3.8 billion metric tons by 2050. This increase highlights the urgent need for improved waste management strategies globally, particularly in rapidly urbanizing regions [15]. Consequently, these countries have introduced measures to control the importation of plastic waste. Some scholars posit that implementing import bans in developing nations could incentivize developed countries to establish new facilities for plastic treatment, although the plastic waste trade remains profitable for traders in the meantime [15].

Among these massive plastic wastes, a considerable amount is directed toward landfills, and some are utilized for energy generation through incineration. However, the incineration process releases hazardous emissions and particulate matter, posing additional environmental and health risks. Moreover, an immense quantity ranging from 10 to 20 million tons of plastics is annually dumped into the oceans, contributing to the gradual deterioration of marine ecosystems [16]. This environmental threat is compounded by the significant consumption of fossil fuels, with about 4 % used as a feedstock for manufacturing plastic products and an additional 3–4% required to fuel these manufacturing industries [17]. Local enterprises in various countries often prefer importing inexpensive plastic waste over investing in domestic waste recycling systems. Plastic waste constitutes over 12 % of the total municipal solid waste (MSW) produced globally, posing a prolonged risk of releasing hazardous chemicals in landfills, potentially leading to groundwater contamination. This risk extends to the presence of microplastic concentrations in the dry sludge disposed of in landfills after wastewater treatment [18]. In some cases, such as in Turkey, the waste management system has been overwhelmed by the swift influx of plastic waste following China's ban, resulting in persistent increases in waste pollution [3,21].

The persistent pollution of the natural environment by plastic waste remains a mounting concern, as it can only be permanently eradicated through destructive thermal treatments like combustion or pyrolysis [22]. Effective waste management strategies, as advocated by the European Union (EU) and the United States Environmental Protection Agency (EPA), prioritize waste prevention and reduction. Among the alternatives, reuse, recycling, and energy recovery are preferred, while disposal is considered the least favorable option. Incorporating biodegradable plastics into these strategies is essential, as they offer new end-of-life waste management options not accessible to non-degradable plastics, such as anaerobic digestion and composting. Biodegradable plastics contaminated with food can undergo composting, unlike non-biodegradable plastics that hinder recycling efforts.

Despite numerous efforts and advancements in plastic waste management, significant gaps remain in implementing sustainable, large-scale solutions. Existing studies primarily focus on conventional methods such as landfilling and incineration, which often exacerbate environmental degradation through pollution and emissions. While recycling and waste-to-energy techniques show promise, their scalability, cost-efficiency, and environmental impact remain areas of concern, especially in regions lacking infrastructure and regulatory support. Additionally, although policies like the European Union's Plastics Strategy and UNEP's Global Commitment to the New Plastics Economy encourage reductions in single-use plastics and sustainable recycling practices, enforcement and cross-regional consistency are ongoing challenges. Also, emerging sustainable technologies for plastic waste management are rarely examined within a comprehensive framework that addresses economic, social, and environmental implications simultaneously. This gap underscores the need for an integrated approach that evaluates both established and innovative practices to inform sustainable policy and practical implementation in diverse socioeconomic contexts. This paper explores how disruptions in plastic waste management can serve as catalysts for both immediate and enduring transformations in global plastic waste management practices. Additionally, it explores selected sustainable technologies for plastic waste management, considering social and economic factors, and addressing challenges in meeting long-term goals.

## 2 Materials and approaches

### 2.1 Literature search strategy

This comprehensive review integrates existing knowledge on the management, challenges, opportunities, and externalities associated with plastic waste for sustainable practices. A systematic search was conducted across multiple academic databases (PubMed, Scopus, Web of Science, and Google Scholar) as well as government and environmental organization reports, using keywords such as “plastic waste management,” “sustainability,” “challenges,” “opportunities,” and “externalities.” Inclusion criteria focused on studies directly related to plastic waste management and sustainability, including peer-reviewed journal articles, conference papers, and reputable reports published from 1990 onwards to capture relevant developments and trends.

### 2.2 Data extraction and analysis

A total of 1150 publications were initially identified. After applying the inclusion and exclusion criteria, 120 publications were selected for in-depth review. The process of data extraction involved systematically condensing essential findings, methodologies, and conclusions from the chosen studies. The collected data were then thematically arranged into sections covering management practices, challenges, opportunities, externalities, and the sustainability implications of plastic waste.

### 2.3 The rationale for dividing literature into eight segments

The literature was categorized into eight segments based on thematic relevance and findings. These segments include: 1. Plastic Generation, which focuses on the production and sources of plastic waste; 2. Projections of Plastic Waste Accumulation by 2060, examining future trends and potential accumulation scenarios; 3. Effect on the Marine Environment, discussing plastic waste impacts on marine ecosystems; 4. Soil Pollution Due to Plastics, exploring effects on soil quality; 5. Air Pollution Due to Plastics, investigating plastic contributions to air pollution; 6. Current Plastic Waste Management Practices, reviewing existing methods for waste management; and 7. Challenges and Prospects, identifying obstacles and future opportunities in waste management. Finally, the review includes suggestions and alternatives for addressing plastic waste issues.

## 3 Results and discussions

### 3.1 Plastic generation

**Fig. 1:**
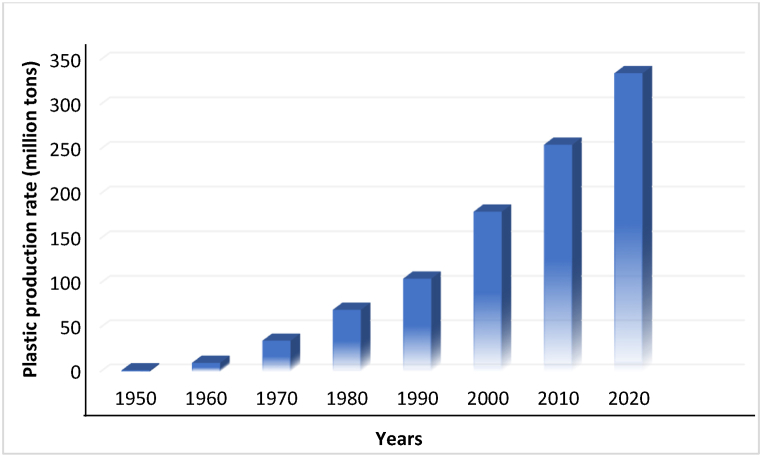
World plastics production 1950–2020.

The heightening Growth of the plastic industry has been visible over than last 50 years. During these periods, Plastics production ramped up from 1.5 million metric tons (Mio. t) in 1950 to ∼322 Mio. t, shown in Fig. 1, and is predicted to increase by more than 600 million tons by 2050. The generation rate gradually increased from 1950 to 1990 and after that, a sharp peak was noticed till 2020. In 1990, plastic production was 105 Mio. t which has increased to 180 million within just 10 years with an increment of about 71 %, which is alarming, and the growth trend is continuous [19]. Global plastic production increased by about 3.4 % in 2015 compared to 2014. The compound Annual Growth Rate (CAGR) of plastic from 1950 to 2015 is about 8.6 % [22].

**Fig. 2:**
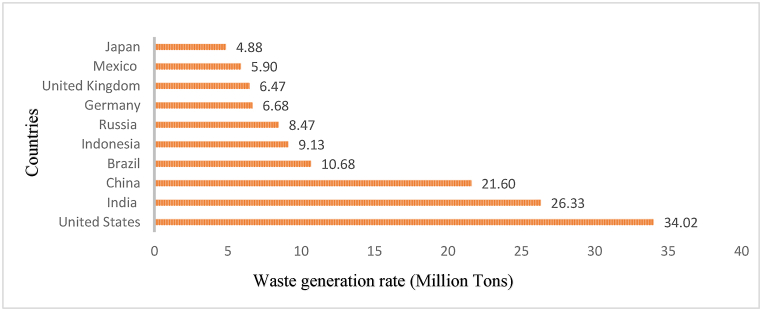
Most plastic waste-generating countries [11].

Plastic has become a part and parcel of lifestyles of people due to its multipurpose nature, outstanding durability, corrosive resistance, good thermal properties, and comparably cheap and non-toxic nature. The distribution of plastic production in the top 10 countries globally is as follows, United States (34.02 Mio. t), India (26.33 Mio. t), China (21.60 Mio. t), Brazil (10.68 Mio. t), Indonesia (9.13 Mio. t), Russia (8.47 Mio. t), Germany (6.68 Mio. t), United Kingdom (6.47 Mio. t), Mexico (5.90 Mio. t), and Japan (4.88 Mio. t) (Law et al., 2020) as shown in Fig. 2. Most waste plastics are generated due to single-use plastics being dumped after their initial application [23].

However, Humans have produced over 8 billion tons of plastic since 1950, with more than half of it ending up in landfills and only about 9 % being recycled [24]. Plastic can be subdivided into two major categories; thermoplastics and thermosetting plastics. Thermoplastics are those that remain flexible under heat. Linear polymers and a combination of linear and cross-linked polymers are examples of thermoplastics, such as PVC, nylon, polythene, etc. However, more populous countries tend to produce more plastic waste overall. The increasing generation of PW is currently a prime concern for waste managers and policymakers as there are very limited facilities to manage this waste.

### 3.2 Projections of Plastic Waste Accumulation by 2060

**Fig. 3:**
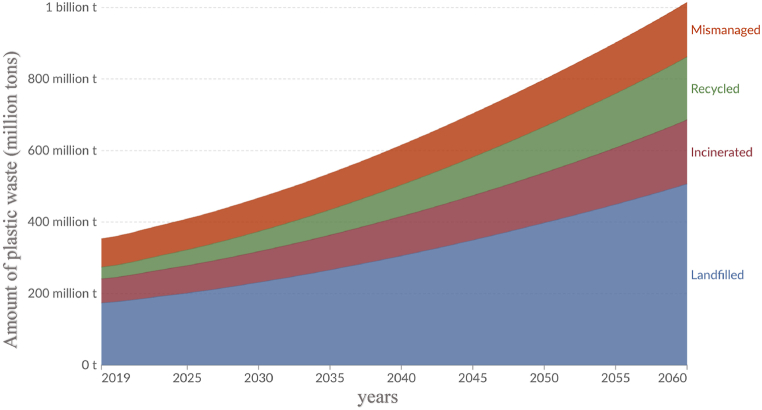
Projections of plastic waste by disposal method (2019–2060). The graph illustrates the projected amounts of plastic waste categorized by disposal methods: mismanaged, recycled, incinerated, and landfilled. Data is based on the 'business-as-usual' scenario, assuming no significant changes in current policies [27].

One widely cited study by Geyer et al. [25] estimates that by 2050, approximately 12 billion metric tons of plastic waste will have been generated, of which 8.3 billion metric tons will have ended up in landfills or the natural environment. This staggering volume of plastic waste poses significant environmental, economic, and social challenges. The plastic production rate is increasing over time due to its diverse applications and advantages, and projections indicate that by 2060, the global plastic production rate could increase by 1100 Mt [26]. However, Fig. 3 provides further insights into how this waste is expected to be managed under a 'business-as-usual' scenario.

According to Fig. 3, plastic waste is projected to reach 1 billion metric tons annually by 2060. Landfilled waste remains the dominant category, accounting for nearly 600 million metric tons by 2060, underscoring the reliance on this disposal method despite its environmental risks. Mismanaged plastic waste, which includes littered or inadequately disposed plastics, is projected to exceed 200 million metric tons by 2060, highlighting a growing threat to ecosystems. Meanwhile, recycling shows modest growth, with an estimated contribution of less than 150 million metric tons by 2060, reflecting insufficient infrastructure and policy focus. Incineration is expected to manage approximately 100 million metric tons annually, but this approach raises concerns about emissions and energy consumption.

If these trends persist, they align with the literature suggesting global recycling, incineration, and discard rates will reach 43 %, 50 %, and 7 %, respectively, by 2050. The Ellen MacArthur Foundation, in collaboration with the World Economic Forum, foresees a troubling scenario in which the quantity of plastic in the oceans could surpass the total mass of fish by 2050 if substantial actions are not taken. The anticipated rise in plastic waste generation, coupled with limited progress in recycling and sustainable disposal methods, underscores the critical need for transformative global action. This emphasizes the urgent need for effective waste management strategies, including robust recycling initiatives and a transition to a circular economy. By embracing sustainable practices and investing in innovative solutions, we can mitigate the environmental and social impacts of plastic waste and work towards a future where plastic waste is significantly reduced.

### 3.3 Health hazards associated with plastic waste

During the manufacturing process, plastics are combined with various additives, such as plasticizers and stabilizers, which pose significant health risks [7]. For instance, bisphenol A (BPA), a plasticizer widely used in polycarbonates and epoxy resins, can interfere with natural hormonal signals, leading to endocrine disorders and other health issues. Beyond the production phase, plastic waste itself presents substantial health hazards, contributing to the pollution of air, soil, and water sources and exposing humans to toxic and carcinogenic substances. Ingestion of microplastics further exacerbates health risks, potentially causing inflammation and gastrointestinal problems. Additionally, air pollution resulting from the incineration of plastic waste introduces respiratory and cardiovascular hazards. Waste workers are also at high risk due to exposure to harmful chemicals during handling and disposal, highlighting the occupational dangers associated with plastic waste management. These combined hazards emphasize the need for effective waste management strategies to mitigate plastic's adverse impacts on both human health and the environment. The properties of different plastic polymers vary widely, influencing their environmental safety and degradation times, as shown in Table 1. For example, LDPE can take between 500 and 1000 years to degrade, while PVC is virtually non-degradable. Certain plastics, such as PETE, PVC, PS, and PP, are especially hazardous due to their leached toxins, which have been linked to cancer, nervous system damage, genetic mutations, and metabolic disorders. These health risks underscore the critical need to regulate plastic production and improve disposal practices to protect human health and maintain ecological balance.

**Table 1:** Plastic types, properties, and associated challenges.

Plastic-type	Time to Decompose (years)	Health hazard	Status	References
PETE	10	Antimony (Carcinogenic)	Not safe	[44]
HDPE	100	estrogen-mimicking chemicals (disrupting hormones)	Usually, safe and low risk	[47]
LDPE	500–1000	estrogen-mimicking chemicals same as HDPE	safe	[19]
PVC	never	BPA, phthalates, lead, mercury	Not safe	[19]
PP	20–30	leaching some chemicals leading to asthma or hormone disruption	Microwave safe	[45]
PS	50	highly toxic, leaching styrene can cause cancer and damage to the nervous system, affect genes	Not safe	[45]

### 3.4 Effect on the marine environment

**Fig. 4:**
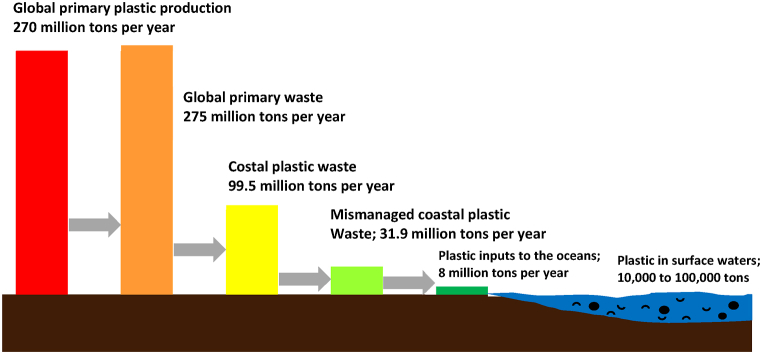
The pathway by which plastic enters the world's oceans [22,30].

The existence of plastics in landfills and oceans poses environmental threats due to the virtually indestructible characteristics of this material. Plastic can inflict gradual yet certain harm on the environment through various means, from releasing toxic chemicals into the soil and groundwater to directly endangering or poisoning animals that unintentionally consume it. For instance, plastic-induced soil pollution has the potential to alter the physical structure of the ground, restricting its ability to retain water. In addition to affecting terrestrial environments, the challenges of plastic waste management often lead to a significant amount of plastic remaining unrecycled and being directly disposed of in landfills. When unsupervised, plastics can degrade into microplastics or become part of the growing waste stream that eventually reaches water bodies. Thus, much of the plastic initially disposed of on land ultimately finds its way into the ocean, where it causes severe ecological disturbances. This pathway through which plastic particles migrate from land to ocean ecosystems is illustrated in Fig. 4.

Once in the ocean, plastic, particularly in the form of smaller particulates, not only affects marine environments physically but also introduces toxic chemicals to the water, impacting marine organisms and ecosystems [28]. Fig. 4 presents the findings of two researchers, with data from 2015 showing that global plastic production rates stood at 270 million tons, with approximately 100 million tons ending up as coastal plastic waste. Among these, mismanaged coastal plastic results in an annual entry of 8 million tons into the ocean. From production, roughly 3 % of plastic ends up in the ocean annually, while the quantity of plastic in surface waters ranges from tens of thousands to hundreds of thousands of tons. In 2021, the Philippines became the largest contributor to ocean plastic, releasing approximately 356,371 tons. These plastics degrade slowly, polluting water bodies and harming marine life. Microplastics have also been detected globally in streams, rivers, lakes, and oceans, affecting ecosystems worldwide [29].

### 3.5 Soil pollution due to plastics

Soil pollution from plastics, especially microplastics, poses major environmental risks. Microplastics—tiny particles smaller than 5 mm—accumulate in soil from various sources and persist over long periods, affecting soil health and ecosystems. Research shows that microplastics can harm soil structure, disrupt nutrient cycling, and reduce the diversity and abundance of soil organisms. Additionally, when soil organisms like earthworms and microorganisms ingest microplastics, they may experience physical harm, behavioral changes, and lower reproductive success [31]. Plastic additives, such as plasticizers and flame retardants, can also leach into the soil, posing toxic risks to soil organisms and further degrading soil health [32]. These impacts disrupt soil food webs and can reduce agricultural productivity. Addressing plastic pollution is essential to protect soil ecosystems and support sustainable agriculture.

### 3.6 Air pollution due to plastics

Plastic-induced air pollution stems from diverse sources, encompassing the production, utilization, and disposal of plastic materials. Plastics contribute to air pollution through processes like manufacturing emissions, incineration of plastic waste, and the disintegration of plastics into the environment. These activities release harmful pollutants and particulate matter into the air, posing substantial health risks. Table 2 summarizes the key pollutants released from plastic-related activities and their effects.

**Table 2:** Key pollutants from plastic-related activities and their effects.

Pollutant	Source	Health Effects	Environmental Impact
**Dioxins and Furans**	Incineration of plastic waste	Carcinogenic, respiratory issues, hormone disruption	Persistent organic pollutants, and bioaccumulation in the food chain
**Volatile Organic Compounds (VOCs)**	Plastic manufacturing and disposal	Respiratory problems, headaches, eye irritation	Contributes to smog formation, impacts air quality
**Particulate Matter (PM**_**10**_**, PM**_**2.5**_**)**	Burning plastics, industrial emissions	Respiratory and cardiovascular diseases	Reduces air quality, affects climate change
**Carbon Monoxide (CO)**	Incomplete combustion of plastics	Headaches, dizziness, impaired cognitive function	Contributes to ground-level ozone formation
**Hydrogen Chloride (HCl)**	Incineration of PVC plastics	Respiratory tract irritation, lung damage	Acid rain formation, soil, and water acidification

Air pollution due to plastics arises from various sources, including incineration of plastic waste, industrial emissions during plastic manufacturing, and open burning. These activities release harmful pollutants into the air, each with specific health and environmental impacts. Dioxins and furans, released during plastic incineration, are highly toxic, causing cancer, hormone disruption, and respiratory issues [20]. Environmentally, they persist in ecosystems and bioaccumulate in the food chain, posing long-term risks to wildlife and humans. Volatile organic compounds (VOCs), emitted during manufacturing and disposal, cause respiratory problems and contribute to ground-level ozone, a component of smog that impacts air quality. Particulate matter (PM_10_, PM_2.5_) from plastic burning and industrial emissions penetrates deeply into the lungs, causing respiratory and cardiovascular diseases, while also impacting climate change [32]. Carbon monoxide (CO) from incomplete combustion can cause cognitive issues and further contributes to ground-level ozone. Hydrogen chloride (HCl), released when incinerating PVC, irritates the respiratory tract and contributes to acid rain, affecting soil and aquatic ecosystems. The widespread use and improper disposal of plastics underscore the need for improved waste management, sustainable alternatives, and stricter regulations on plastic incineration to protect health and the environment.

The production of plastic materials releases volatile organic compounds (VOCs) and greenhouse gases (GHGs) during raw material extraction, polymer synthesis, and energy-intensive processes, contributing to air pollution and climate change with respiratory and cardiovascular health risks [33]. Additionally, the incineration of plastic waste emits hazardous pollutants like dioxins, furans, heavy metals, and polycyclic aromatic hydrocarbons (PAHs), linked to respiratory and immune disorders, and recognized as carcinogenic and endocrine-disrupting agents [34]. Over time, plastics degrade into microplastics, which can become airborne, carrying chemical additives and absorbing other pollutants, further impacting air quality and human health [35]. Reducing plastic use, promoting recycling and circular economy practices, and endorsing sustainable alternatives, along with cleaner production technologies, are essential for reducing emissions and protecting health and the environment.

### 3.7 Current plastic waste management practices

Post use, most of the plastics become PWs, and to manage these wastes, several strategies have been adopted globally. There are two strategies to manage PW which include landfilling, and recycling [33]. Landfilling is the least preferred technique in plastic waste management (PWM) due to its detrimental environmental impact on soil and groundwater [36]. Landfilling is the oldest technique for PWM which was previously adopted by most countries [37]. However, the process is unsuitable as PW can remain unchanged without biodegradation for more than 1500 years [38]. Thus, currently developed countries putting strategies and discouraging the landfilling action of plastic.

**Fig. 5:**
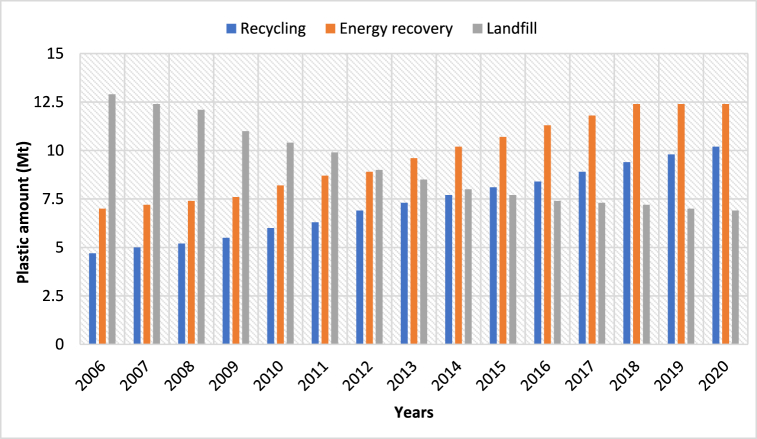
Recycling, Energy recovery, and Landfill rate from 2006 to 2020.

Fig. 5 illustrates the trends in plastic waste management from 2006 to 2020, highlighting the interplay between recycling, energy recovery, and landfilling. Over this period, recycling has seen a gradual increase, growing from approximately 2 Mt in 2006 to nearly 5 Mt by 2020, reflecting advancements in recycling infrastructure and efforts to promote circular economy practices. Energy recovery, which involves converting plastic waste into energy, shows a sharper rise, increasing from 4 Mt in 2006 to over 7 Mt by 2020, surpassing recycling as a favored method [47]. While energy recovery offers an alternative to traditional disposal, its reliance on incineration raises environmental concerns, particularly related to emissions. Landfilling, the most dominant method in earlier years, accounted for over 12 Mt in 2006 and peaked near 13 Mt in 2015 before declining to approximately 10 Mt by 2020. This decline suggests a shift in waste management priorities, with efforts aimed at reducing reliance on landfills due to their long-term environmental impacts, such as leachate formation and methane emissions. Overall, while recycling and energy recovery have grown, the total volume of plastic waste managed has steadily risen, highlighting the need for more sustainable strategies to reduce plastic generation and enhance recycling efficiency.

However, recycling is a good option for PWM to use the limited resources and minimize the impacts on the environment. The recycling process includes primary recycling, secondary recycling (mechanical recycling), tertiary recycling (chemical recycling), and quaternary recycling (energy recovery) [39]. Recycling involves the process of collecting, sorting, processing, and converting plastic waste into new products or materials. It helps conserve resources, reduce energy consumption, and minimize the carbon footprint associated with plastic production [40]. Furthermore, the Mechanical process involves the conversion of plastic waste into raw materials, known as plastic regrinds or flakes. These materials can be used as feedstock in the production of new plastic products. Mechanical recycling is suitable for plastics that can be melted and reprocessed without significant degradation, such as polyethylene terephthalate (PET) used in beverage bottles and high-density polyethylene (HDPE) used in various packaging. Additionally, the chemical recycling (CR) process involves the conversion of plastic waste into raw materials, known as plastic regrinds or flakes. These materials can be used as feedstock in the production of new plastic products. Mechanical recycling is suitable for plastics that can be melted and reprocessed without significant degradation, such as PET used in beverage bottles and high-density polyethylene (HDPE) used in various packaging [48]. Results from the graph shows, recycling is getting popular with time and in the period 2006–2020, the recycling rate almost doubled and heightened continuously. Finally, quaternary recycling, also known as the energy recovery process, has recently gained popularity. There are several methods for converting plastics into energy, the most common of which are hydrothermal liquefaction (HTL), gasification, and pyrolysis [41]. PW can be converted into valuable products using these techniques. By embracing and enhancing recycling efforts, we can significantly reduce the environmental burden of plastic waste and move towards a more sustainable and resource-efficient future.

Additionally, Incineration remains a common method for plastic waste management, particularly in regions with limited landfill space or advanced waste-to-energy infrastructure. Incineration can reduce plastic volume significantly and generate energy, but it also raises environmental concerns due to the release of harmful emissions, including dioxins, furans, and heavy metals, which pose health risks and contribute to air pollution [34,44]. Moreover, while incineration was once a commonly used method for managing plastic waste, it is increasingly recognized for its negative environmental effects and is now less frequently employed in many countries. Incineration involves the controlled combustion of plastic waste at high temperatures, typically ranging from 850 °C to 1200 °C. This process is widely used in various countries to manage plastic waste efficiently.

Table 3 shows the details of incineration rates in developed countries. For instance, Japan leads globally with an incineration rate of 80 %, utilizing advanced technologies to convert waste into energy, which contributes significantly to their energy supply and reduces landfill dependency. In Sweden, where incineration accounts for 50 % of waste management, facilities are integrated into district heating systems, further enhancing its sustainability impact [50]. Germany, with a 35 % incineration rate, emphasizes strict emission control standards in its facilities to mitigate environmental impacts effectively [51]. In contrast, the USA, with a lower incineration rate of 12 %, faces challenges due to ample landfill availability and stringent regulatory requirements [52]. Despite its benefits in reducing waste volume and generating energy, concerns persist over emissions like dioxins and particulate matter, necessitating continuous advancements in emission control technologies to align with environmental sustainability goals globally. In countries such as Japan and some European nations, incineration is carefully regulated with advanced technology to capture pollutants, making it a viable part of waste-to-energy strategies [31]. However, in areas with less stringent environmental controls, incineration can exacerbate environmental issues, especially when plastics are burned without adequate pollution control measures [17]. While incineration is effective for waste volume reduction, its long-term environmental impact highlights the need for alternative methods, such as recycling and material recovery, which can mitigate emissions and promote circular economy practices [45].

**Table 3:** Incineration rates and practices in selected countries.

Country	Incineration Rate (%)	Description
**Japan**	80 %	Japan leads in incineration technology, with many facilities generating energy from waste.
**Sweden**	50 %	Sweden uses incineration for waste-to-energy, contributing significantly to district heating systems.
**Germany**	35 %	Germany's incineration plants comply with strict emission regulations, reducing environmental impact.
**USA**	12 %	In the USA, incineration is less common due to landfill availability and regulatory challenges.

### 3.8 Challenges and prospects

To address and manage this substantial volume of plastic waste, plastic managers, policymakers, and concerned authorities are undertaking various measures. One potential approach is to reduce the use of single-use plastic, given that a significant portion of these items tend to be discarded into the environment after their initial use. However, this phenomenon can be complex if the general public and policymakers aren't working hand in hand together. The market for single-use plastics is very large due to the high usage of plastics in food packaging and other industries. The government needs to take the strong initiative to decrease or ban the single use of plastics. However, the recycling rate needs to be increased. The ratio of plastic manufacturing to recycling rate is comparatively very low. There are several reasons behind it and the cost of recycling plastic is higher than newly manufactured plastic. The government needs to take proper initiatives to solve these issues by encouraging the manufacturers to recycle the plastics and bounce them with proper opportunities [42].

**Fig. 6:**
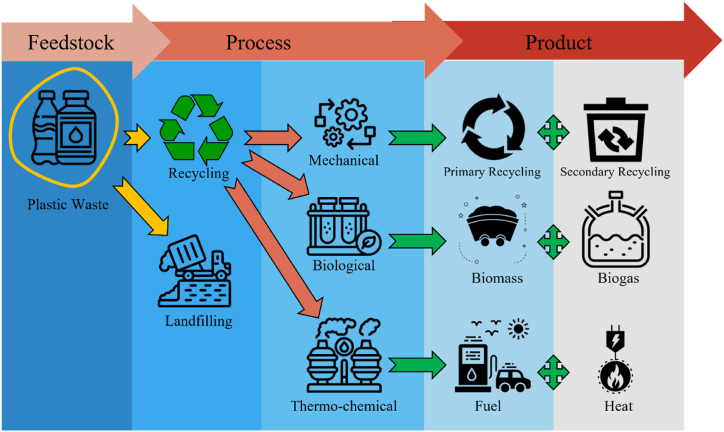
Routes of plastic waste-from generation to end product.

Fig. 6, a large quantity of waste plastic is going through the recycling process. Efforts to improve recycling infrastructure, technology, and consumer education can lead to higher recycling rates for plastic waste. This can reduce the amount of plastic sent to landfills and incinerators. Advancements in sorting technologies, such as optical scanners and robotics, can enhance the efficiency and accuracy of plastic waste sorting, making recycling more effective.

There are three well-known recycling techniques for plastic waste: mechanical, biological, and thermo-chemical conversion processes. Each technique has distinct methods and outcomes, contributing to a comprehensive approach to plastic waste management. A detailed overview of plastic waste management techniques has been illustrated in Table 4.

**Table 4:** Overview of plastic waste management techniques.

Technique	Process Description	Benefits	Challenges	Environmental Impacts	Potential Alternatives
**Mechanical Recycling**	Physical processing of plastic waste into new products without altering the chemical structure.	Reduces plastic waste; reuses plastic materials.	Quality degradation after multiple cycles; sorting challenges.	Reduces raw material demand; lower carbon footprint.	Advanced sorting technologies; improved material design.
**Biological Recycling**	Breakdown of plastic waste using biological agents like bacteria, fungi, or enzymes.	Decomposes biodegradable plastics; and produces non-toxic end products.	Limited to biodegradable plastics; slow process.	Minimal toxic residue; reduces landfill use.	Development of more biodegradable plastics.
**Gasification**	Heating plastic waste to high temperatures (above 700 °C) in the presence of controlled oxygen or steam, producing syngas (hydrogen, carbon monoxide, carbon dioxide).	Converts plastic waste into syngas for power generation and chemical production.	High energy requirements; complex technology.	Emissions of greenhouse gases; potential pollutants.	Integration with renewable energy sources; emission controls.
**Pyrolysis**	Heating plastic waste in the absence of oxygen at temperatures between 300 °C and 900 °C, producing pyrolysis oil, char, and syngas.	Converts plastic waste into oil, char, and syngas; processes mixed plastic waste.	High energy requirements; complex technology.	Reduces landfill waste; and emissions of volatile organic compounds (VOCs).	Scaling up; improving efficiency.
**Hydrothermal Liquefaction**	Applying high pressure and moderate temperatures (200 °C–374 °C) in the presence of water to convert plastic waste into liquid hydrocarbons.	Produces crude oil-like substances and aqueous phase products; refines into transportation fuels.	High energy requirements; water use.	Potential water contamination; emissions.	Water treatment technologies; energy integration.
**Landfilling**	Disposing of plastic waste in landfills.	Low initial cost; convenient for large volumes of waste.	Limited space; long-term environmental impact.	Soil and groundwater contamination; greenhouse gas emissions.	Improved waste segregation; landfill mining and reclamation.

Mechanical recycling involves the physical processing of plastic waste into new products without altering the chemical structure of the material. The process includes collecting, sorting, washing, shredding, and melting the plastic waste, which is then reformed into pellets or flakes. These pellets can be used to manufacture new plastic products. Mechanical recycling is widely used for materials like PET and HDPE (high-density polyethylene), commonly found in beverage bottles and containers [53]. While mechanical recycling helps reduce plastic waste, it has limitations, such as the degradation of plastic quality after multiple recycling cycles and the challenge of sorting different types of plastics.

Biological recycling, also known as biodegradation, involves the breakdown of plastic waste using biological agents like bacteria, fungi, or enzymes. This method is particularly suitable for biodegradable plastics, which are designed to decompose more quickly than traditional plastics [54]. Biological recycling can occur under controlled conditions in industrial composting facilities or in natural environments. The end products of biological recycling are typically water, carbon dioxide, and biomass, which can be used as soil conditioners. However, this technique is still under development for many types of plastics, and its efficiency depends on the environmental conditions and the specific microorganisms used.

Moreover, thermo-chemical conversion processes involve the chemical transformation of plastic waste into valuable products through the application of heat and chemical reactions. Key thermo-chemical processes include gasification, pyrolysis, and hydrothermal liquefaction. Gasification involves heating plastic waste to high temperatures (above 700 °C) in the presence of a controlled amount of oxygen or steam, resulting in the production of syngas (a mixture of hydrogen, carbon monoxide, and carbon dioxide) [55]. Syngas can be used as a fuel for power generation or as a feedstock for producing chemicals and fuels. Pyrolysis heats plastic waste in the absence of oxygen at temperatures between 300 °C and 900 °C, breaking down the plastic into smaller molecules [56]. The end products include pyrolysis oil (which can be refined into diesel or other fuels), char (used as a solid fuel or carbon source), and syngas. Pyrolysis is versatile and can process mixed plastic waste, making it a promising method for recycling non-recyclable plastics. Hydrothermal liquefaction involves the conversion of plastic waste into liquid hydrocarbons by applying high pressure and moderate temperatures (200 °C–374 °C) in the presence of water [49]. The resulting products include crude oil-like substances, which can be refined into transportation fuels, and aqueous phase products that can be treated for further use. From these thermo-chemical conversion processes, valuable energy products such as biogas, heat, and transportation fuels can be obtained, providing a dual benefit of waste reduction and energy production.

However, despite these recycling techniques, a significant portion of plastic waste still ends up in landfills, where it can persist for hundreds of years, leaching harmful chemicals into the soil and water. This underscores the need for improved waste management practices, increased recycling rates, and the development of sustainable alternatives to reduce the environmental impact of plastic waste. The prospects of plastic lie in our collective efforts to embrace sustainable practices, adopt innovative technologies, and implement effective policies. By transitioning to a circular economy model, we can minimize plastic waste, conserve resources, and protect our environment. The journey towards a sustainable future for plastic requires collaboration among stakeholders, including governments, industries, researchers, and consumers. Together, we can shape a future where plastic coexists harmoniously with our planet.

### 3.9 Possible alternatives

**Fig. 7:**
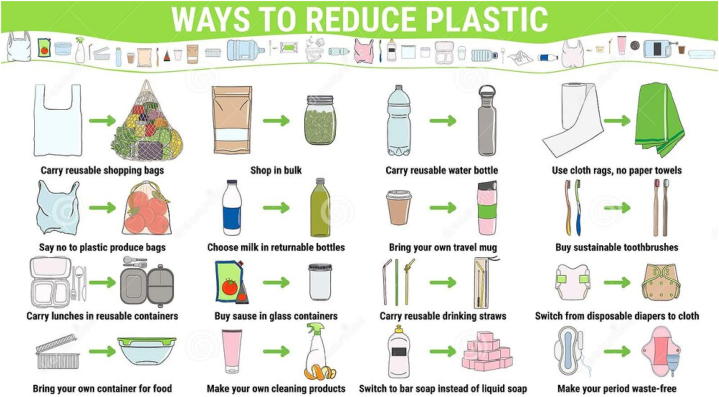
Alternatives of single-use plastics (Hall et al., 2006).

In light of the environmental impacts of plastic waste, a variety of sustainable alternatives offer promising solutions. Fig. 7 illustrates various alternatives to single-use plastics that can contribute to a more sustainable future. These alternatives can be organized into three main types: Reusable Materials, Biodegradable Options, and Sustainable Packaging. Each category includes materials and practices designed to reduce plastic reliance and support environmental conservation.

Reusable materials provide durable, long-lasting alternatives to single-use plastics, helping to reduce waste by encouraging repeated use. Stainless steel and glass are highly durable materials used in bottles, jars, and containers. Metal water bottles and glass storage containers are widely used for food and drink, as they can be reused multiple times without degrading. Natural materials like bamboo, wood, and ceramics offer eco-friendly replacements for plastic in household items. Bamboo utensils, cutting boards, and even toothbrushes are popular options due to bamboo's rapid growth rate and biodegradability. Cotton, jute, and hemp are versatile natural fibers used for bags and packaging. Cotton and jute bags are especially popular as reusable shopping bags, providing an alternative to single-use plastic bags [40]. Using these reusable materials minimizes the environmental impact by reducing plastic waste and conserving resources.

Biodegradable materials decompose naturally, minimizing long-term environmental impact. They are especially useful in single-use items that can break down safely after disposal. Derived from renewable sources such as corn starch and sugarcane, bioplastics decompose much faster than traditional plastics and are commonly used for packaging, utensils, and disposable items in the food industry. Innovative materials like algae and biopolymers are used to create plant-based, biodegradable water bottles that decompose in a natural environment. These bottles offer a sustainable alternative to traditional plastic bottles. Biodegradable polymers, often used in food wrappers and sachets, provide environmentally friendly packaging options that prevent long-term waste accumulation [43]. Biodegradable options are particularly beneficial in the food and beverage industries, as they reduce waste and encourage compostable practices.

Sustainable packaging focuses on reducing plastic use in packaging materials by utilizing renewable, recyclable, or compostable materials. Paper, cardboard, and bamboo are commonly used in packaging and are easily recyclable, providing eco-friendly options that replace plastic equivalents. Metal containers, silicone storage bags, and cloth shopping bags offer durable, reusable alternatives to single-use packaging, with silicone food bags and stainless-steel lunch boxes especially popular for food storage due to their longevity and ease of cleaning. New developments like jute polymer bags, eco-friendly poly bags, and paper bottles aim to replace conventional plastics in packaging applications. These materials offer a sustainable solution by being compostable or recyclable, reducing the environmental footprint of packaging waste.

Fig. 7 highlights the importance of adopting practical alternatives to reduce plastic pollution, conserve natural resources, and promote sustainable consumption and production practices. By integrating these alternatives into daily routines, individuals and communities can play a pivotal role in combating the growing plastic waste crisis. Actions such as using reusable shopping bags, carrying refillable water bottles, and switching to eco-friendly products like bamboo toothbrushes or cloth diapers not only reduce reliance on single-use plastics but also inspire a culture of responsibility and mindfulness [46]. These small, incremental changes collectively contribute to reducing the burden on landfills, preventing plastic leakage into oceans, and conserving valuable resources for future generations. Furthermore, adopting practices like buying in bulk, making homemade cleaning products, and transitioning to a waste-free menstrual cycle align closely with global sustainability goals such as those outlined in the UN's Sustainable Development Goals (SDGs). Ultimately, this Fig. 7 underscores that achieving a significant reduction in plastic waste requires a shift in both individual behavior and systemic practices, emphasizing the shared responsibility of all stakeholders in fostering a healthier, more sustainable environment.

## 4 Conclusion

In conclusion, the global surge in plastic waste generation presents substantial environmental and ecological challenges. While an immediate reduction in plastic production is difficult due to its widespread use, strategic initiatives can significantly mitigate the negative impacts. Key measures include developing alternative plastic sources such as biodegradable materials, reducing reliance on single-use plastics through bans and restrictions, and utilizing thermochemical processes to convert plastic waste into energy. These efforts align with Sustainable Development Goals, particularly SDG 12 (Responsible Consumption and Production) and SDG 14 (Life Below Water), promoting a cleaner and safer environment. Expanding local recycling industries is essential to effectively manage plastic waste, requiring robust infrastructure and widespread adoption of recycling practices. Collaborative efforts from governments, policymakers, social activists, and citizens are crucial to drive these initiatives forward. Public awareness campaigns and innovative technologies can further enhance these efforts, leading to a significant reduction in plastic waste. By fostering a collective commitment to sustainable practices and embracing innovative solutions, we can address the plastic waste crisis. This concerted action will not only mitigate environmental pollution and associated health issues but also steer the world towards a more sustainable future, ensuring a cleaner, greener, and healthier planet for generations to come.

## Data availability

Data is available from the corresponding author upon reasonable request.

## Declaration of competing interest

The authors declare that they have no known competing financial interests or personal relationships that could have appeared to influence the work reported in this paper.
